# Considerations for addressing bias in artificial intelligence for health equity

**DOI:** 10.1038/s41746-023-00913-9

**Published:** 2023-09-12

**Authors:** Michael D. Abràmoff, Michelle E. Tarver, Nilsa Loyo-Berrios, Sylvia Trujillo, Danton Char, Ziad Obermeyer, Malvina B. Eydelman, Foundational Principles of Ophthalmic Imaging and Algorithmic Interpretation Working Group of the Collaborative Community for Ophthalmic Imaging Foundation, Washington, D.C., William H. Maisel

**Affiliations:** 1 Departments of Ophthalmology and Visual Sciences, and Electrical and Computer Engineering, University of Iowa, Iowa City, IA USA; 2 Center for Devices and Radiological Health, US Food and Drug Administration, Silver Spring, MD USA; 3 OCHIN, Portland, OR USA; 4 Center for Biomedical Ethics, Stanford University School of Medicine, San Francisco, CA USA; 5 Department of Anesthesiology, Stanford University School of Medicine, Division of Pediatric Cardiac Anesthesia, San Francisco, CA USA; 6 School of Public Health, University of California, Berkeley, CA USA

**Keywords:** Health policy, Medical ethics

## Abstract

Health equity is a primary goal of healthcare stakeholders: patients and their advocacy groups, clinicians, other providers and their professional societies, bioethicists, payors and value based care organizations, regulatory agencies, legislators, and creators of artificial intelligence/machine learning (AI/ML)-enabled medical devices. Lack of equitable access to diagnosis and treatment may be improved through new digital health technologies, especially AI/ML, but these may also exacerbate disparities, depending on how bias is addressed. We propose an expanded Total Product Lifecycle (TPLC) framework for healthcare AI/ML, describing the sources and impacts of undesirable bias in AI/ML systems in each phase, how these can be analyzed using appropriate metrics, and how they can be potentially mitigated. The goal of these “Considerations” is to educate stakeholders on how potential AI/ML bias may impact healthcare outcomes and how to identify and mitigate inequities; to initiate a discussion between stakeholders on these issues, in order to ensure health equity along the expanded AI/ML TPLC framework, and ultimately, better health outcomes for all.

## Introduction

The US Department of Health and Human Services defines health equity^1^ as the absence of avoidable disparities or differences among socioeconomic and demographic groups or geographic areas in health status and health outcomes such as disease, disability, or mortality^2^. While there are multiple reasons for avoidable health inequities^2^, lack of equitable access to diagnosis and treatment are prominent in diseases ranging from breast cancer, depression, to diabetic eye disease^3–8^. Fostering health equity has been a goal of healthcare stakeholders: patients and their organizations, providers, ethicists, payors, regulators, legislators, and AI creators. With the exponential growth in new digital health technologies and the rise of artificial intelligence/machine learning (AI/ML)-enabled medical devices, innovators may potentiate existing disparities or instead, leverage opportunities to mitigate health inequities^9^.

Artificial Intelligence (AI) systems can perform tasks that mimic human cognitive capabilities^10^, or may perform new functions that humans are unable to do^11^. Such AI systems are typically not explicitly programmed, the systems learn from data that reflect highly cognitive tasks that may otherwise be performed by trained healthcare professionals. In many cases, AI systems are intended to aid healthcare professionals (HCPs) in managing or treating patients; there are also AI systems intended to be used directly by patients to help manage a disease or condition^12^. Healthcare AI systems have the potential to foster access to healthcare for underserved populations, while improving care quality at both the level of the individual patient and the population, at reduced cost for patients, payors, and society^2–8,10,12–16^.

Some healthcare AI-enabled devices have been authorized by FDA and have been in clinical use for over a decade, with more devices being currently developed. While the vast majority of AI systems intended to be used by HCPs serve to aid those HCPs, there are also AI/ML-enabled devices that make a clinical decision without human oversight, including the first point-of-care autonomous AI system on the US market^17^, which received national coverage and reimbursement thereby allowing widespread deployment^18,19^. Thus, AI systems are increasingly in a position to help improve patient and population health outcomes and drive down cost, increase physician job satisfaction, and address health disparities^20,21,3,22,23^.

However, adding AI to healthcare processes may unintentionally have undesired effects. Multiple studies have shown examples of the use of AI in healthcare (not evaluated by regulatory agencies) exacerbating, rather than mitigating, health disparities^24,25^. This is especially the case where the systems that utilize AI do not adhere to rapidly emerging evidence-based standards^26^, or where these may be designed for non-marketed use but ultimately are used more broadly. One study of a widely-used AI system showed that while its stated goal was to identify patients who needed extra help with their complex health needs, its actual objective function (its “achieved goal”) was to predict healthcare costs. This use of the AI system out of context resulted in sicker, Black patients receiving similar care to healthier, White patients despite needing higher acuity care. Thus, the inherent bias in the algorithm appeared to contribute to worse outcomes for Black patients by influencing the likelihood they would receive the appropriate level of care^25^.

These ethical and other concerns with AI in healthcare have been shown by a number of research studies^27–29^. Abramoff et al. proposed ethical frameworks for AI^27–31^ to help proactively address the issue of undesirable algorithmic bias as well as other concerns with AI. More recently, the Foundational Considerations on Algorithmic Interpretation (FPOAI) workgroup of the Collaborative Community on Ophthalmic Imaging published their “Foundational Considerations”^32^ on AI as a start to developing *metrics for ethics*, including metrics for “Equity”^33^, in order to be able to evaluate how specific AI systems adhere to various bioethical principles.

### Bias in the healthcare process

Undesirable bias (“bias” in short) in the conceptualization, development and application of AI-ML-enabled medical devices that is not acknowledged or addressed has the potential to exacerbate existing health inequities or create new disparities. In its recent *Artificial Intelligence/Machine Learning (AI/ML)-Based Software as a Medical Device (SaMD) Action Plan*^10^, FDA articulated the importance of addressing bias in the development and use of AI/ML-enabled medical devices.

Healthcare is the prevention, treatment, and management of medical conditions and the preservation of mental and physical well-being^34^. Through a series of processes and medical products implemented or delivered by healthcare providers, improved outcomes can be realized for patients and populations. Opportunities exist for AI alone or in combination with healthcare providers, to deliver healthcare solutions. The use of AI is rapidly expanding, and examples of AI that have been implemented include assistive AI for breast cancer screening, hypertension management, stroke management, and autonomous AI for diabetic eye exams. Ethical frameworks that consider the potential negative and positive implications of widespread collection, analysis and use of large datasets can be used to determine whether a given healthcare process meets the goal of achieving good health outcomes for all patients. Typically, a *Pareto optimum* is sought between multiple bioethical principles, such as beneficence, autonomy and equity (“Justics”)^32^. How much a process meets a specific bioethical principles, can be quantified using ‘metrics for ethics’, and these may affect benefit-risk determinations. While it is beyond the scope of these “Considerations” to exhaustively list these metrics, we give some examples to illustrate the principle. For the principle of beneficence, it can be a common metric such as sensitivity, specificity, or clinical outcome, while for the principle of equity, it can be sensitivity disaggregated by demographic subgroup, differential clinical outcome across subgroups, or even population achieved sensitivity or specificity to measure the impact of access to the process, as we have defined previously^32,35^.

The focus of these “Considerations” is to determine how much a given healthcare process (that may include AI) meets the equity principle. To illustrate, a given process may improve healthcare outcomes for a patient, or a population, on average. However, when we consider outcomes across the population in more detail, this assumed improvement through integration of the AI system may not be evenly distributed across the population, though disease characteristics including prevalence, severity and prognosis may be otherwise equally distributed. The AI may affect a large variance in outcome improvement for some groups compared to others, so that some groups may have substantially worse outcomes than others, such as in the example mentioned above^25^.

### Measuring bias in AI systems

Bias in any part of the healthcare process can lead to differential impacts on different groups^36^, and historically has resulted in poorer health outcomes for underrepresented, underserved, and under-resourced groups^1^. Examples of such groups are groups that are defined by racial, ethnic, age, sex, gender, national origin, disability, religion, political, or genetic information characteristics^1^. Thus, such bias reduces the bioethical principle of “Justice,” as Char et al.^28^ and Abramoff et al.^32^ described. On the basis of such ethical frameworks, and the continuing development of “metrics for ethics,” bias can be quantified as differential impact of a healthcare process on a particular group. Humans delivering healthcare can also exhibit bias; for example, a recent study showed provider bias, where providers’ charts documented Black patients’ symptoms and signs in a more pejorative manner, with the potential to exacerbate health disparities^37^. Other studies suggest physician bias in caring for other populations as well^38–40^.

How much any process, whether partially delivered or aided by AI, or fully delivered by humans, meets the “equity” bioethical principle can be quantified in various ways. Such measurements are necessarily specific to the use case and patients’ risk of harm being considered, but an emerging set of studies draws on new data to measure algorithmic performance. In fact, such measurements have shown that AI systems can counter bias by human decision makers^11^. Similarly, in a diagnostic process, there may be concern about equity in accuracy. Subgroup statistical testing for the presence or absence of an effect on diagnostic accuracy (“accuracy disaggregation”) could be used to determine how well the equity principle is met^32,41^. As an example, where there is concern about access to a diagnostic test*, population-achieved sensitivity and specificity*, which measures the impact of both access and sensitivity, has been proposed as a way to understand the impact of bias on population health, when including so-called invisible populations^32^. By allowing optimization of population-achieved sensitivity and specificity, this metric can aid in improving population outcomes through diagnostic assessment, including those performed by AI.

Ultimately, understanding and mitigation of AI bias starts with assessment and quantification of possible sources of bias along the entire lifecycle of an AI device. Identification of bias is only part of mitigation, and stakeholders will have to decide, based on the AI context and perceived benefit/burden ratios, the extent to which identified biases can and should be mitigated.

### The AI total product lifecycle

Bioethical analysis of the AI lifecycle by Char et al.^28^ highlighted the pipeline, ranging from conception over development to deployment (“access”) of AI systems, and the parallel pipeline of evaluation and oversight activities at each stage. On top of this model, we analyzed the key factors associated with ethical considerations, from the existing literature as well as newly identified. This pipeline model framework is useful for systematic ethical appraisals of AI systems from development through deployment, and for interdisciplinary collaboration of diverse stakeholders that will be required to understand and subsequently manage the ethical implications of AI in healthcare^28^. Abramoff et al. subsequently linked this model to specific metrics for the conception, design, development, training, validation and implementation phases of AI technologies in healthcare^32^.

Another approach to decompose AI bias into different components has been proposed^42^. The approach divides sources of algorithmic bias into three main components: (direct) model bias, training data variance and training data noise. However, this approach, which focuses on AI built exclusively from retrospectively collected data, incorrectly assumes that the reference standard (sometimes referred to as *ground truth*) to compare the AI system to is perfectly correct^32^, and focuses solely on the potential for bias in the AI/ML algorithm. It does not consider the other ‘pipeline phases’ as set forward by Char et al. nor the integration of the AI into the care process. In other words, it does not consider what matters most to patients and other stakeholders: whether or not the addition of an AI system to the care process results in a favorable change in care – i.e., improved clinical outcome^43,32^. Gianfrancesco et al. similarly limited their analysis to bias derived from characteristics of retrospective training sets derived from existing Electronic Health Record (EHR) data^44^.

The framework developed by Char et al., on the other hand, recognizes specific AI system pipeline phases: conception, development, calibration, implementation, evaluation, deployment, and oversight, and the ethical considerations, including “equity” along each of these phases.

In 2019, FDA illustrated how the Total Product Lifecycle (TPLC) approach to the regulation of medical devices similarly applied to AI systems that meet the definition of a medical device, in its *Proposed Regulatory Framework for Modifications to Artificial Intelligence/Machine Learning*^45^. TPLC describes the different phases of a device, including software such as AI systems, from conceptualization to its impact once on the (US) market as the following:

- Conception,
- Design,
- Development,
- Validation,
- Access and marketing, and
- Monitoring.

These TPLC phases map in a straightforward manner to the ‘pipeline phases’ as defined in Char et al.^28^ and as operationalized in Abramoff et al.^32^, see also ref. ^46^. Thus, our intent is to extend TPLC to ethical analysis, by considering the potential impact on equity at each of these phases, as well as the potential for mitigation of AI bias, as defined above. From an ethical perspective, the equity principle can be analyzed and optimized within each phase of the existing TPLC framework. Depending on which TPLC phase is considered, standard equity metrics can be added, such as for the development phase, key performance indicators in software development, quality systems and risk of population harm analysis, or, for the validation phase of diagnostic medical devices, and absence of racial or ethnic effects on sensitivity and efficacy^32^.

**Fig. 1:**
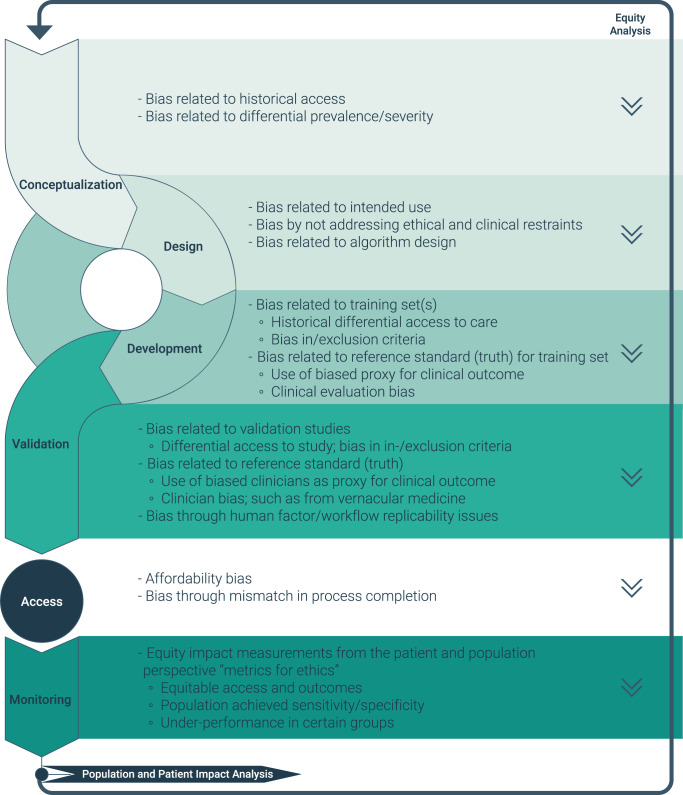
Total Product LifeCycle (TPLC) equity expanded framework with examples for each phase.

The proposed TPLC framework in Fig. 1 adds equity considerations for AI systems, including the wider context of where the AI system is used in healthcare, with the goal of net benefit for the entire target population^35^. This framework (Fig. 1) is not intended to be comprehensive for all bias risks and mitigations, however it does initiate a discussion on AI and healthcare equity along the TPLC. The present framework is intended to complement the principles outlined in the International Medical Device Regulators Forum’s (IMDRF)^47^ Software as a Medical Device Clinical Evaluation, FDA’s Good Machine Learning Practice^48^ the aforementioned documents, but specifically hone in on ways to identify and mitigate bias in the development and evaluation of AI-ML-enabled software. It helps illustrate the importance of proactively developing an analytical framework to aid in identifying sources of impactful bias along the TPLC before a proposed AI tool propagates health disparities.

As shown in Fig. 1, there is the potential to favorably, or unfavorably, impact health equity at every phase of the TPLC. Phases differ in the nature of potential bias, as well as it and its effects on health equity can be quantified and mitigated. Importantly, the equity impact at each phase is independent of all other phases, in other words, even when all potential bias has been mitigated in earlier phases, the next phase can still introduce undesirable equity effects. While Fig. 1 is not exhaustive, it highlights the opportunities to consider equity and bias along the TPLC. Thus, equity, considered upstream in the development process, has potential ripple effects on the downstream health outcomes.

### Conception phase

At conceptualization of the AI/ML-enabled medical device, it is critical to think through the TPLC development paradigm and identify opportunities to address and mitigate bias. During the conception phase, consider the health conditions and the care process in which the AI system will be used. Determining which health condition(s) will be the focus of the AI/ML-enabled medical device, may at the outset be directed at fostering health equity. Technologies that target conditions where the burden of disease is shouldered by a specific segment of the population may lead to more opportunities for improving health outcomes in that population. Examples might include developing an AI/ML-enabled device that helps diagnose narrow angle glaucoma or open angle glaucoma, conditions with higher prevalence in Asian, or Black and Latino populations, respectively. In addition, it may be important to determine the setting in which the AI/ML-enabled device will be used at the outset to help identify and mitigate bias. This requires optimization between generalizability and scalability of an AI/ML algorithm on the one hand, and on the other hand, optimizing its development, training and validation for the populations in which it will be used. For example, if an AI system for a diagnostic process is developed, trained, and validated only on those with ready access to healthcare services, but intended and deployed as a screening tool for an entire community or population, some of who lack routine healthcare access, the differential healthcare access may be a major source of inequity and AI-induced bias. The impact of such differential access can be measured for example through population-achieved sensitivity^32^ compared to overall sensitivity, i.e., the fraction of correctly identified disease cases in a sample, without regard to representativeness of that sample (or lack thereof).

Additionally, historical data used in the development of AI/ML-enabled devices may be fraught with miscategorized, mislabeled or mis-tagged, and missingness that differentially impacts different segments of the population. For example, historically reported similarities in disease phenotype, prevalence, or severity across groups may not reflect the actual differences in disease phenotype, prevalence or disease severity across groups. This bias may result from historical differences in access to care, differential treatment and quality of care offered in the healthcare system^49^, as well as differing group concerns about data usage and ownership^50^. Such incorrect assumptions about the disease under study may lead to incorrect, biased AI systems from conceptualization^51^. Abramoff et al. asserts such bias may also be the logical result of ‘vernacular medicine’ which are regional biases in care that may not expand to broader communities^32^. The inclusion of various viewpoints, backgrounds, experience and expertise on the creator team (including engineers, data scientists, clinicians, and other AI creators) may be an additional opportunity to avoid or mitigate the continuation of such biases into “vernacular AI” during each phase of the TPLC.

### Design phase

During this phase, consider the equity implications related to intended use of the medical device. In addition to the health condition for which the device will be used, other aspects of intended use including the operators and needed skills to use the device (e.g., human factors/usability engineering); the ways in which the device will integrate into the clinical workflow, the length of time needed to effectively use the device and the associated burden on patients and providers; the target patient population; and disease spectrum can all impact utilization and broad access to the technology. Not addressing the ethical and clinical constraints that were described in the conception stage may result in solidifying bias in the AI design. AI validity, explainability and transparency all help assess the equity implications of the Software as a Medical Device’s (SaMD)’s algorithm design^32^. The introduction of using racially invariant priors instead of fully deriving the algorithm from training data may be one approach to prevent the introduction of AI bias^41^. The TPLC model is foundational to how FDA regulates medical devices, and Design and Development phases are typically rapidly iterated: we emphasize that biases introduced during each of these two phases will propagate to the other, if not mitigated before the next iteration.

### Development phase

In the development phase of the AI algorithm, training dataset selection is another opportunity to proactively include equity considerations. Initial considerations for the training sets used in AI/ML-enabled devices were published by FDA in 2021^10^, and further expanded in the Good Machine Learning Practice (GMLP) document^48^. It is important to consider whether the relevant characteristics of the intended patient population (such as age, gender, sex, race, and ethnicity), use, and measurement inputs are sufficiently represented in training and test datasets, to maximize generalization to the intended population in which the AI system will be used^48^. Bias issues may arise around a) retrospective use of historical datasets b) more or less inclusive contemporary or prospectively collected datasets, and c) clinical study verification (covered in Validation section). For example, use of historical datasets may reflect differential access to care and differential quality of care due to sociocultural forces may lead to skewed distribution in the training data^50^. Prospective collection of data for training datasets is not exempt from potential biases. The eligibility criteria or other aspects of the recruitment and enrollment process, such as the reward or time commitment for data collection (e.g., need to miss work) could potentially be a constraint for people with limited financial resources – the so-called “invisible populations”. Finally, bias in the ‘reference standard’, for the training dataset, may be caused by using inadequate proxies for clinical outcomes as reference standard^32,52^. For considerations around which reference standard to use, see Abramoff et al. 2021^32^ As an example, if clinicians are used as the reference standard, their potential bias in their diagnosis may lead to bias in the training data, ultimately persisting as bias in the AI system^37^. Similarly, outcomes or proxies thereof used as reference standards may reflect historical inequities for subgroups, so that access and bias in delivery of care for subgroups may result in differential outcomes for the same disease phenotype. Metrics for such training set bias may be assessed through subgroup analysis and stratification of characteristics.

### Validation phase

Important factors to consider in the validation phase for AI/ML-enabled devices have been included in documents, such as the International Medical Device Regulators Forum (IMDRF’s) Software as a Medical Device (SaMD): Clinical Evaluation^53^, and more recently in FDA’s Guiding Principles for Good Machine Learning Practice (GMLP)^48^. We use the term *validation* consistent with how it is used within the context of medical device development, i.e., ‘confirmation by examination and provision of objective evidence that the particular requirements for a specific intended use can be consistently fulfilled’^52,54^. When considering bias in validation, it is critical to evaluate how well clinical study subjects are mirrored in the data sets on which the AI system was conceptualized, designed, and developed. Ensuring that the relevant characteristics of the intended patient population including age, gender, sex, race and ethnicity, are appropriately represented in a sample of adequate size in a clinical study, allows results to be reasonably generalized to the intended use population. Thoughtful evaluation will expose bias and enhance appropriate and generalizable performance across the intended patient population. In addition, diversity in clinical sites where the studies are conducted will be an important consideration to generate diverse validation studies. Historically disadvantaged groups may be more likely to receive care in clinics that may lack the resources for the trained operators necessary to be a study site^55^. By considering metrics for how similar to real-world use the trial is (e.g., metric for operator expertise and diagnosability), there may be an opportunity to expand inclusion of more diverse clinical sites in the trial. These approaches can be assessed for their impact on replicability of findings in other samples of patients such as whether preregistration and arm’s length protocols are followed^32^.

### Access and monitoring phases

The access and monitoring phase includes deployment, monitoring and surveillance of the AI/ML-enabled device’s performance and may also be subject to bias in implementation. This phase is where we have an opportunity to more comprehensively consider and measure the cumulative effects of potential biases at all phases of the TPLC, with real world evidence. In other words, we can estimate whether the ‘real-world realization’ of the AI system as it was originally conceptualized, designed and developed, measurably impacts health equity. During this phase, creators can assess the a priori vision of how well the AI-enabled device fits into the clinical workflow, and is usable with the prespecified staff skills, usability, cost and other resource use^32^. For example, if monitoring shows that low resourced patients are unable to access the device because the clinics in those locations cannot afford the high cost of the device, such as in under-resourced, or rural clinics, then the goals of the AI-enabled device to impact health outcomes in this population may not be realized; this can be quantified by a metric like population achieved sensitivity^10^. This monitoring information may thus lead to re-conceptualization of the device, for example with lower cost hardware, and more sophisticated ML algorithms to increase accessibility of the device in these populations^56^. Receiving care with the AI system may also impart higher cost or higher copay for the patient which may impact patients’ access differentially. AI-induced bias can be introduced here through mismatch or shifts in process completion. For example, a process that combines identifying and treating true cases of diabetic retinopathy in people with diabetes may be skewed towards negative outcomes if there is differential follow-through for treatment. This follow-through for treatment is also subject to the same social determinants of health that can lead to inequitable utilization of healthcare services, and thereby lead to biased assessments of the device’s performance. While these factors are best considered in the concept phase, effects on equity can be quantified and mitigations implemented here. Metrics such as population achieved sensitivity and specificity, device underperformance in certain groups, and other metrics of equitable access and outcomes can be assessed across subgroups longitudinally, and may help determine at what stage of the TPLC there may be opportunities to mitigate inequities. The above shows the importance of monitoring AI’s impact in the real world, and the limitations of current frameworks for how to think about monitoring and surveillance in such a real-world setting: discussion among all stakeholders is crucial.

## Discussion

We describe the sources and impacts of bias in AI systems on health equity, and propose approaches for potential mitigation across the AI’s Total Product Lifecycle (TPLC). These Considerations are the start of a discussion with all stakeholders, including bioethicists, AI creators, regulatory agencies, patient advocacy groups, clinicians and their professional societies, other provider groups, and payors and value-based care organizations. Equity analysis and bias mitigation consistent with the present, expanded TPLC, will allow AI creators, regulators, payors and healthcare practitioners to better understand how potential bias may impact healthcare decisions and outcomes. The many potential sources of bias that can be introduced or addressed along the different phases of the TPLC can be assessed using appropriate metrics and mitigated using tailored approaches. By focusing on the goal of ensuring health equity along the TPLC framework, stakeholders can collectively identify and mitigate inequities, leading to better health outcomes for all.

### Reporting summary

Further information on research design is available in the Nature Research Reporting Summary linked to this article.

### Supplementary information

Reporting Summary
